# Slipped capital femoral epiphysis: an epidemiological Nationwide study in Italy from 2001 to 2015

**DOI:** 10.1186/s12891-021-04435-x

**Published:** 2021-06-22

**Authors:** Umile Giuseppe Longo, Rocco Papalia, Sergio De Salvatore, Laura Ruzzini, Vincenzo Candela, Ilaria Piergentili, Leonardo Oggiano, Pier Francesco Costici, Vincenzo Denaro

**Affiliations:** 1 Department of Orthopedic and Trauma Surgery, Campus Bio-Medico University of Rome, Rome, Italy; 2 Department of Surgery, Orthopedic Unit, Bambino Gesù Children’s Hospital, Rome, Italy

**Keywords:** Epiphysiolysis, Slipped capital femoral epiphysis, SCFE, In situ fixation, Dunn procedure, Triplane proximal osteotomy, Epidemiology, Surgery, Young

## Abstract

**Background:**

Slipped capital femoral epiphysis (epiphysiolysis of the femoral head, SCFE) is the most common pediatric hip disease in 10–14 years old children. The most used procedure to correct a stable form of SCFE is in situ pinning. Instead, the proper treatment for unstable forms is controversial. The first purpose of this study was to estimate annual admissions for SCFE in Italian patients from 2001 to 2015, basing on the hospitalization reports. The second aim was to assess the difference between regions regarding SCFE procedures. Lastly, a statistical prediction of the volume of SCFE procedures performed in Italy based on data from 2001 to 2015 was performed.

**Methods:**

Data of this study were collected from the National Hospital Discharge Reports (SDO) reported at the Italian Ministry of Health regarding the years of this paper. The yearly number of hospital admission for SCFE, the percentage of males and females, the average age, days of hospitalization, primary diagnoses and primary procedures in the whole Italian population were calculated using descriptive statistical analyses.

**Results:**

From 2001 to 2015, 4893 hospitalizations for SCFE were recorded in Italy, with a mean incidence of 2.9 (cases/100.000 inhabitants). The majority of patients treated by SCFE were males (70.6%).

**Conclusion:**

National health statistics for SCFE are attractive for an international audience, as different approaches to screening are reported between countries. These differences allow comparing outcomes internationally. Moreover, sharing national statistics and correlating those to other countries protocols, could be helpful to compare outcomes for different procedures internationally. However, further studies are required to understand the specific reasons for regional variation for SCFE procedures in Italy.

**Level of evidence:**

III

## Background

Slipped Capital Femoral Epiphysis (also known as Epiphysiolysis of the femoral head, SCFE) is the most common pediatric hip disease that affects patients 10–14 years old [1, 2]. SCFE is defined by posterior and inferior displacement (through the epiphyseal plate) of the proximal femoral epiphysis with the metaphysis. Each year in the USA, approximately 10.8 cases per 100.000 children of SCFE occurs [3, 4], and 18–50% are bilateral [5]. A timely diagnosis is challenging due to the relative frequency of the disease and the lack of significant symptoms [6]. Pain along with the adductor muscle (groin pull is uncommon in adolescents) is the most frequent symptom. The aetiology is multifactorial (endocrine diseases, hypogonadism, panhypopituitarism, growth spurts), but pediatric obesity represents the most relevant risk factor [7, 8]. SCFE is classified (using radiography and clinics) as stable or unstable forms based on the stability of the femoral physis and the capability to weight-bearing [9]. The former is treated using in situ closed screw fixation; instead, open reduction and fixation are usually adopted for unstable forms [10]. However, the proper treatment for unstable forms is still debated [11–13]. The major problem of this disease is the rapidity of diagnosis and the timing of surgery. The decision between conservative or operative treatment could also be influenced by geographical factors [14]. The prevalence of SCFE surgery in Europe is not fully defined, and only Sweden reported the nationwide incidence of this disease [15]. National health statistics for SCFE are attractive for an international audience, as different approaches to screening are reported between countries (type of screening, method of classification, mean age at the time of screening, diagnosis and subsequent treatment protocols). These differences allow comparing outcomes internationally. Moreover, sharing national statistics and correlating those to other countries protocols, could be helpful to compare outcomes for different procedures internationally.

This study was conducted from 2001 to 2015, based on official data source as hospitalization records. The principal purpose is to evaluate the yearly number of SCFE surgeries in Italy. The second purpose is to assess geographical variation in hospitalization for SCFE between three macro-areas of Italy (North, Center and South). Finally, a statistical projection of the volume of SCFE procedures in the next 5 years was performed.

## Methods

Data of this study were collected from the National Hospital Discharge Reports (SDO) reported at the Italian Ministry of Health regarding the years of this paper (2001–2015). These reports provided data concerning all hospital admission occurring in Italy, both from public and private institutions. In Italy, the regional authorities are responsible for organizing and supervising healthcare services delivered through local structures (public or private). Data on the healthcare services are collected by hospitals and periodically sent to the Ministry of Health [16]. These data are anonymous and described the patient’s age, sex, residence, the region of hospital admission, days of stays, diagnoses and procedures [17]. Population data were obtained from the National Institute for Statistics (ISTAT) for each year [18]. Epiphysiolysis was defined by the following International Classification of Diseases, Ninth Revision, Clinical Modification (ICD-9-CM) with the diagnosis code: 732.2. Since SCFE procedures for patients over 19 years were only 263 over the 15-year study period, the study was restricted to the patients with 0–19 years of age to avoid underestimation.

### Macro-areas of Italy

**Fig. 1:**
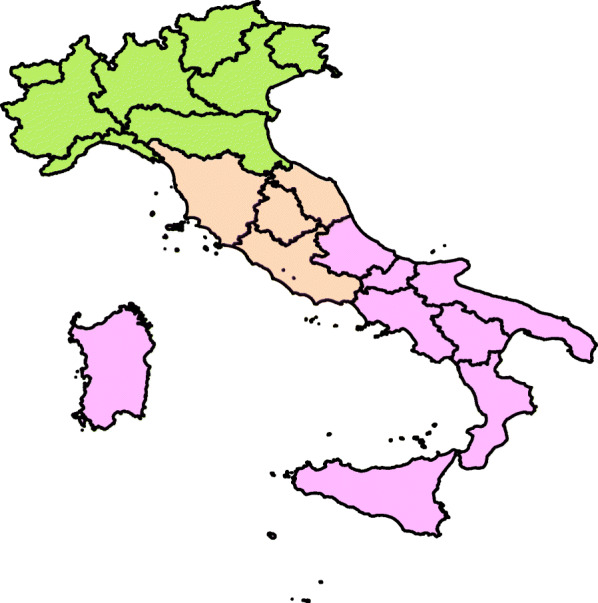
Macro-areas of Italy are the North, the Centre and the South. (The figure was made using R software version i368 4.0.3)

Italy is divided into three macro-areas: North, Center and South-and-islands. The former includes four regions in the Western part (Aosta Valley, Liguria, Lombardy and Piedmont) and five in the Eastern part (Autonomous Province of Trento, Autonomous Province of Bolzano, Friuli-Venezia Giulia, Emilia-Romagna and Veneto). The Center counts four countries (Lazio, Marche, Tuscany and Umbria). The latter part of Italy includes five regions (Apulia, Basilicata, Calabria, Campania and Molise) and the islands (Sardinia and Sicily) (Fig. 1).

### Population

The authors divided the patients for macro-area of domicile (North, Centre and South). The patients were divided into two groups (named “regional” and “extra-regional surgeries”) to identify the presence of possible migratory flux. The former includes patients that were treated in the same macro-area of their domicile. Instead, the extra-regional group included patients who migrated from their domicile and were treated in other macro-areas.

### Statistics

The authors used a descriptive statistical analysis were estimate the yearly number of SCFE procedures, the percentage of males and females, the average age, days of hospitalization, primary diagnoses and primary procedures in Italy. The annual adult population size obtained from ISTAT, a statutory electronic national population register, was used to calculate the incidence rates [19]. The incidence was based on the size of the whole Italian population of patients under 19 years old. To find statistical differences between years or sex, the linear regression analysis and the Mann-Whitney U Test were used, as applicable. The Exponential Smoothing (ETS) algorithm without seasonality was used to assess the forecast model. The Statistical Package for Social Sciences (SPSS) version 26 was used for this data analysis. Tables, graphs and forecast were performed using Excel (Microsoft) software.

## Results

### Population

**Fig. 2:**
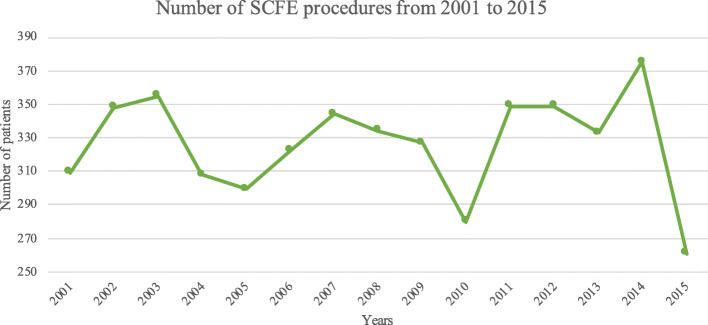
Number of SCFE procedures from 2001 to 2015

During the study period, 4893 SCFE procedures were performed in Italy (Fig. 2), representing an incidence of 2.9 procedures for every 100,000 Italian inhabitants (0–19 years old). From 2001 to 2015, the incidence of operations slight decreased from 2.8 to 2.3 per 100,000 person-years 0–19 years old without statistically significant result (*p* = 0.5) (Table 1).

**Table 1:** Incidence of SCFE procedures per 100′000 resident from 2001 to 2015

Years	Frequency	Percent	Incidence
**2001**	309	6.3	2.8
**2002**	348	7.1	3.2
**2003**	355	7.3	3.2
**2004**	308	6.3	2.8
**2005**	299	6.1	2.7
**2006**	322	6.6	2.9
**2007**	344	7.0	3.0
**2008**	334	6.8	2.9
**2009**	327	6.7	2.9
**2010**	280	5.7	2.4
**2011**	349	7.1	3.1
**2012**	349	7.1	3.1
**2013**	333	6.8	2.9
**2014**	375	7.7	3.3
**2015**	261	5.3	2.3
**Total**	4893	100.0	2.9

**Fig. 3:**
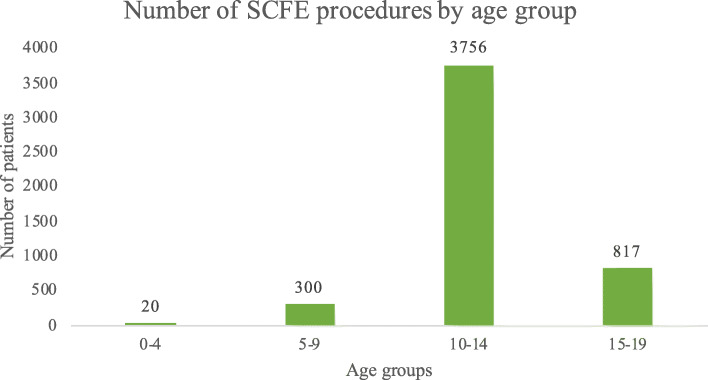
Number of SCFE procedures by age group

Over the study period, the 10–14-year age group reported the highest incidence of surgeries (Fig. 3).

**Fig. 4:**
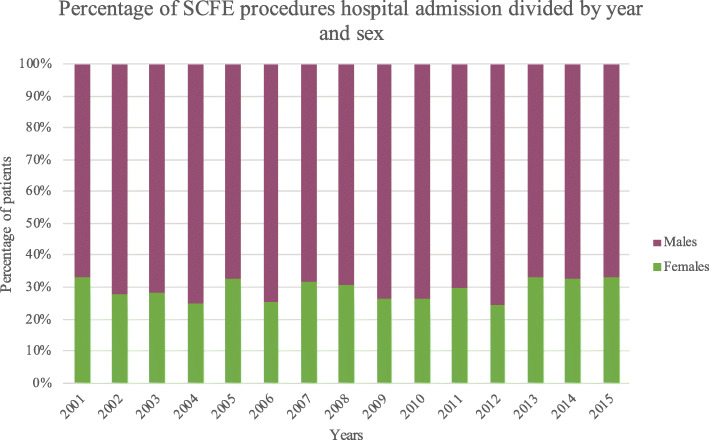
Percentage of SCFE procedures hospital admission divided by year and sex

Males represented the majority of patients, both in total and over the years (females 29.4% and males 70.6%) (Fig. 4). No statistically significant differences in sex trend during the years were found (*p* = 0.4).

**Fig. 5:**
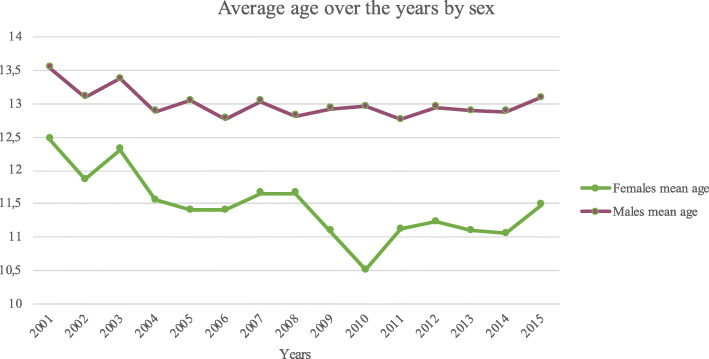
Average age over the years by sex

From 2001 to 2015, the average age of patients was 12.55 ± 2.2. During the entire period, the average age of males was always higher than in females (mean age of females 11.5 ± 2 and mean age of males 13 ± 2; *p* < 0.001) (Fig. 5).

### Days of hospitalizations

**Fig. 6:**
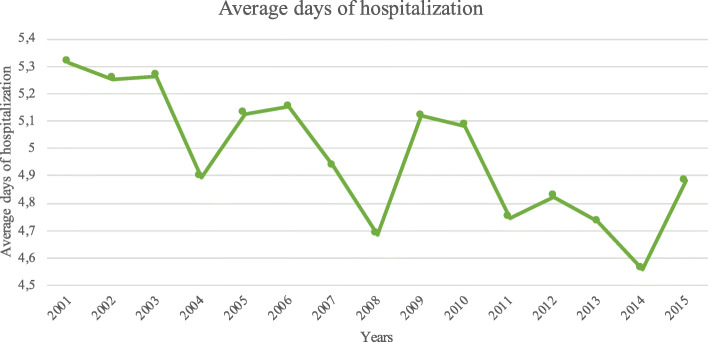
Average days of hospitalization during the years

The mean length of days of hospitalization was 4.97 days (range 1–62 days). Males had more days of admission than females (M: 4.97 and F: 4.95 days; *p* = 0.8). A general trend of decrease in days of hospitalization in both groups was observed (*p* = 0.001) (Fig. 6).

**Fig. 7:**
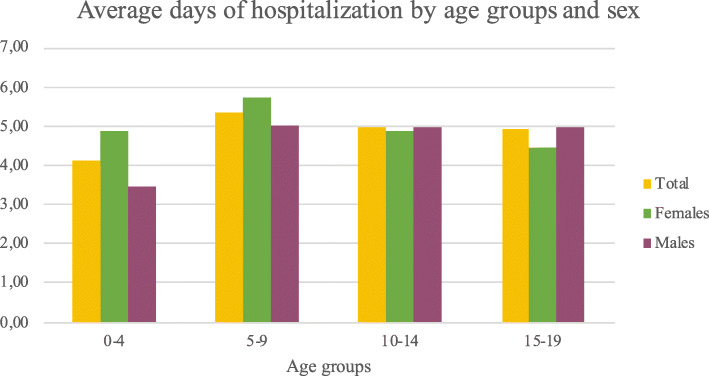
Average days of hospitalization by age groups and sex

Patients aged from 5 to 9 had more days of hospitalization on average. Differentiating by sex, males with a higher number of days of hospitalization are between 10 and 14 years old, while women between 5 and 9 years old (Fig. 7).

### Region of admission and migratory flow

Regarding the regional distribution, 2422 cases of SCFE procedures were performed in the North (49.5%), 868 (17.7%) in the Center, and 1603 (32.8%) in the South. Of 4893 SCFE procedures performed in Italy during the study period, data on the patient’s domicile was not available for 20 patients; therefore, only 4873 procedures were included in the analysis. From 2001 to 2015, 1491 patients (30.6%) lived in the North, 823 patients (16.9%) in the Center, and 2559 patients (52.5%) in the South. Regional surgeries were 99.1% in the North, 77.9% in the Center and 61.9% in the South. The highest rate of extra-regional surgeries was recorded for patients that moved from the South to the North (29.7%) and from the Center to the North (21.0%). All the data are reported in Table 2.

**Table 2:** Analysis of migratory flows by macro-region 2001–2015

Analysis of migratory flows by macro-region 2001–2015			
**Macroregion of residence**	**Macroregion of hospitalization**	**Frequency**	**Percent**
**Unspecified**	*Center*	5	25.0
	*North*	10	50.0
	*South*	5	25.0
	***Total***	**20**	**100.0**
**Center**	*Center*	641	77.9
	*North*	173	21.0
	*South*	9	1.1
	***Total***	**823**	**100.0**
**North**	*Center*	8	0.5
	*North*	1478	99.1
	*South*	5	0.3
	***Total***	**1491**	**100.0**
**South**	*Center*	214	8.4
	*North*	761	29.7
	*South*	1584	61.9
	***Total***	**2559**	**100.0**

### Procedure performed and admission diagnosis codes

**Fig. 8:**
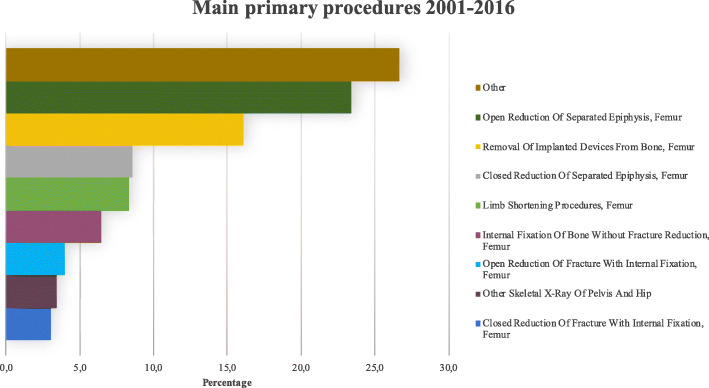
Main primary procedures for SCFE from 2001 to 2015

During the 15-year study period, the main primary diagnoses were “Nontraumatic slipped upper femoral epiphysis” (84.1%), “Aftercare involving internal fixation device” (3.8%), “Other orthopaedic aftercare” (3.6%) and “Encounter for removal of internal fixation device” (2.4%). The main primary procedures performed are reported in Fig. 8.

### Projection model

**Fig. 9:**
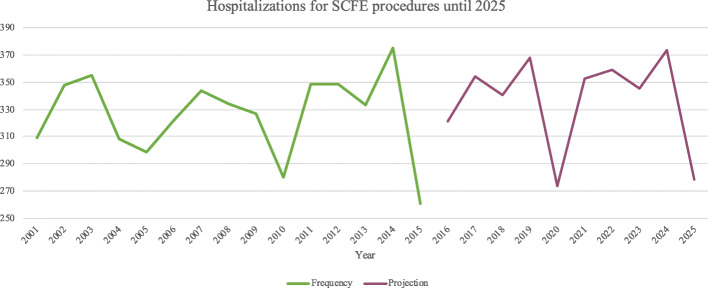
The projection model showed a stable volume of hospitalizations for SCFE procedures in the next 5 years

The projection model indicates a stable volume of hospitalization for SCFE procedures (Fig. 9). The projection model showed that the demand for SCFE procedures hospital admissions was estimated to remain unchanged from 261 in 2015 to 278 by 2025.

## Discussion

Epiphysiolysis of the femoral head is the most common hip disease in the pediatric population [13, 20], with an incidence of 0.2–0.3 per 100,000 children aged 10–14 years [1]. The aetiology of SCFE is multifactorial and includes endocrine disorders, growth spurs and obesity [2, 4, 7, 21–23]. History of trauma to the hip is uncommon [9]. The most common symptoms are pain and limping localized to the hip, groin, thigh or knee [24, 25]. A precise and rapid diagnosis is challenging due to the differential causes of hip pain in young patients [11]. Apophyseal avulsion fracture or apophysitis of the anterosuperior and anteroinferior iliac spine; septic arthritis and adductor muscle strain need to be excluded in these patients [4, 26]. Moreover, also transient synovitis, fractures and Legg-Calvè-Perthes should present similar symptoms. However, these conditions are uncommon in the SCFE age group [4, 26]. A delayed diagnosis could avoid short and long-term complications as avascular necrosis (AVN) of the femoral head and hip osteoarthritis, respectively [27]. Symptom’s duration is used to classify SCFE in acute, acute on chronic and chronic forms. If symptoms present within 3 weeks, it is considered acute; instead, after 3 weeks, it is chronic [28]. The former is the most dangerous because related to a higher rate of AVN. Loder and colleagues [9] classified the stability of the physis based on the patient’s capacity to bear weight, with or without crutches. Moreover, it is possible to evaluate SCFE by radiographical parameters using the Wilson and Southwick methods [9, 29, 30]. The initial step in the treatment of SCFE is to place the patient on non-weight bearing crutches or in a wheelchair [31]. It is mandatory to prevent slip progression and the insurgence of complications [32]. A closed reduction should not be attempted because it can result in AVN caused by the restricted blood supply to the epiphysis [33, 34]. Some authors recommend the prophylactic treatment of the contralateral hip, but there is no consensus concerning this topic [35]. There is a lack of high-quality literature on SCFE surgical management. However, based on the current literature, the best treatment for stable SCFE is in situ pinning with a single screw, performed regardless of the timing of presentation [36]. The unstable SCFE is related to a higher risk of osteonecrosis (20–50% of cases) [37–39], but the proper treatment and the timing associated with the lowest risk of AVN are still debated [11, 36].. The technique described by Parsch and colleagues (open capsulotomy and partial reduction) seems to be the most promising, reporting a low rate of AVN [40]. Moreover, the modified Dunn procedure historically reported satisfactory outcomes with a low rate of necrosis, but it is widely influenced by the surgeon’s technique and skills [36].

The most relevant complications of SCFE are AVN; degenerative osteoarthritis; acute loss of cartilages known as chondrolysis (reported after SCFE surgery or in untreated SCFE); femoroacetabular impingement [3, 12, 41–43]. The rate of AVN varies in the literature, but it is usually more frequent in unstable SCFE compared to stable forms [36]. Loder and colleagues [9] reported an AVN incidence of 0 and 47% for stable and unstable SCFE forms, respectively. However, recent literature reported an incidence of AVN between 0 and 3.3% for stable forms and 23.9% for unstable forms [32].

SCFE is a relevant disease in the pediatric population and deserves to be known by clinicians. The main diagnosis code used for this analysis was 732.2 (Epiphysiolysis). The main procedures performed for SCFE were the “Open Reduction Of Separated Epiphysis, Femur” (23.4%), followed by “Limb shortening Procedures, Femur” (8.3%). In Italy, from 2001 to 2015, the mean incidence of hospital admission for SCFE was 2.9 for every 100,000 Italian inhabitants 0–19 years old. The majority of patients were males of the 10–14 years age group, in line with the Swedish results, as reported by Herngren et al. [15]. Males reported a higher mean age compared to females (*p* < 0.001). The highest number of patients treated was domiciled in the South of Italy (*n* = 2559), followed by the North (*n* = 1491) and the Center (*n* = 823). Otherwise, the highest number of procedures were performed in the North (*n* = 2422) and the South (*n* = 1603). The highest number of “extra-regional surgeries” were patients from the South that migrated to the North or the Center. Instead, patients from the North and the Center tended to be hospitalized in their macro-region of domicile. Moreover, patients from the South reported a higher rate of diagnosis of epiphysiolysis compared to the North and the South. A significant decrease in days of hospitalization during the study period was found, but further studies are required to identify possible explanations. The forecast model showed that the demand for SCFE hospital admissions was estimated to remain unchanged from 2015 to 2025.

To our knowledge, the only study on the SCFE surgery trend was performed by Herngren et al. [15]. However, only patients aged from 9 to 15 years were included, while a broader range of age was analysed in the present paper (0–19 years old). Moreover, the study period considered by Herngren et al. [15] was shorter compared to the present study (from 2007 to 2013 vs 2001 to 2015, respectively).

### Limitations

This study is based on administrative data from different hospitals and macro-regions. The International Classification of Diseases 9 (ICD-9) was used for all the procedures reported. Otherwise, with the ICD-9, it was possible to use different codes for the same surgical procedure. This heterogeneity of codification could lead to an underestimation of our results. Secondly, the database has not been subject to internal validation. Moreover, we found 16.1% of “Removal of Implanted Devices from Bone, Femur” within the procedures included. This data could lead to an overestimation of our results concerning the migratory flux and the total amount of procedures performed. Patients over 19 years old (*n* = 263) were excluded to avoid underestimation. Moreover, it was impossible to distinguish monoliteral vs bilateral screw fixation because ICD-9 did not fully code it. Lastly, this is a database study, and therefore it is not possible to define specific reasons for migratory flux. Further studies are required to define this trend precisely.

## Conclusions

The incidence of surgery for SCFE in Italy is 2.9 cases/100,000 inhabitants of the same age group (from 2001 to 2015). The higher rate of hospitalization for SCFE was recorded in the South and the North. Epidemiological studies are helpful to understand the national variation of a specific surgical procedure and compare them with other countries. However, further studies are required to understand the specific reasons for regional variation for SCFE procedures in Italy.

## Data Availability

The datasets used and/or analyzed during the current study are available from the corresponding author on reasonable request. The access to the database is on request. All data were obtained by the Direzione Generale della Programmazione Sanitaria—Banca Dati SDO of the Italian Ministry of Health.
